# Lennart Philipson (1929–2011): A Warrior Has Passed

**DOI:** 10.1371/journal.pbio.1001153

**Published:** 2011-09-20

**Authors:** David Baltimore

**Affiliations:** California Institute of Technology, Pasadena, California, United States of America

**Figure:**
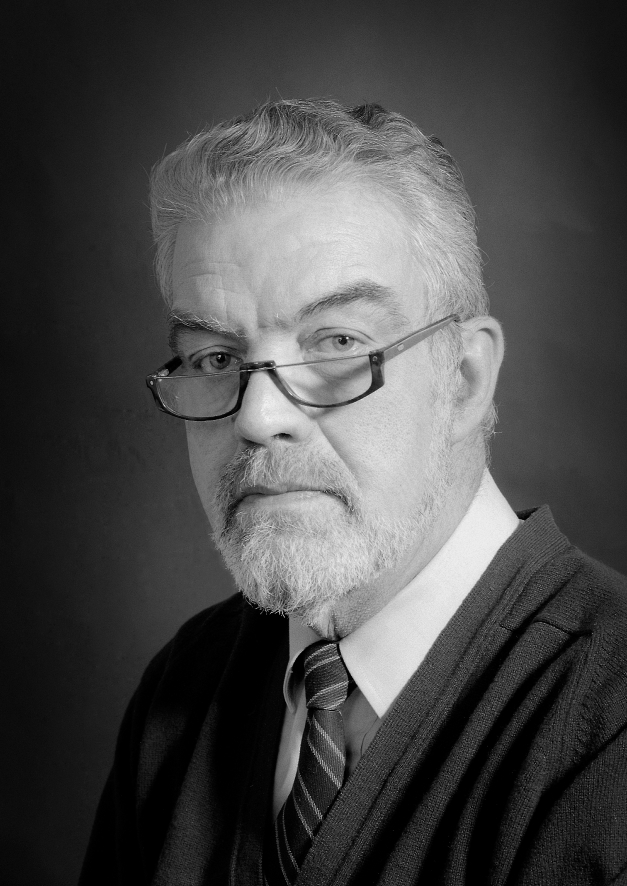
Lennart Philipson. Image credit: European Molecular Biology Laboratory.

Lennart Philipson died on June 26, 2011. His passing is a great loss to the worldwide biomedical research community. He was a big man—physically, emotionally and operationally, dominating rooms, psyches and institutions. He was my wonderful friend, and when Lennart became your friend, it filled a big need in your life.

Lennart was a Swedish virologist/microbiologist who earned both an MD and a PhD in 1957–1958, the latter for work on respiratory viruses. He then went to the Rockefeller University for post-doctoral work in virology, returning after a couple of years to the Wallenberg Laboratory in Sweden's Uppsala University, where he established his own laboratory. It happened that I entered the Rockefeller University just after Lennart left and did my thesis work on the same floor where he had worked. You could still feel his presence. So many ice buckets had his name on them, and if you paged back in the centrifuge log, it was all Philipson. People talked about his energy, his intensity, and his formidable persona. I was excited to meet him on a return trip to the United States and quickly saw why he was so unforgettable.

Virology has enjoyed a golden era over the past 50 years, largely because advances in molecular methods could be rapidly applied to viruses to uncover remarkable molecular details. Lennart flourished in this environment, quickly rising to prominence as he concentrated first on picornaviruses and then on adenoviruses. Always staying au courant with the latest technology, Lennart was poised to ride each new wave of technological advance as it unfolded. He is particularly noted for his work on the cellular receptors for viruses, on the assembly of adenoviruses, and on the control of adenovirus gene expression, but he made contributions to fields as disparate as serology and structure determination and everything in between. He later studied genes that inhibit cellular growth.

Lennart did a sabbatical in my laboratory and quickly made himself at home: soon I wondered if he or I was running the show. He orchestrated a very important set of experiments, showing that synthesis of the reverse transcriptase in murine retroviruses comes about by suppression of a termination codon. This work solved the vexing puzzle of how, from one open reading frame, a major and a minor product could emerge. I had a chance to see up close what a force of nature he could be in the laboratory.

Lennart was not only an important scientist, he was also a great institutional builder and administrator of science. By 1967 he had become the Director of the Wallenberg Laboratory and went on to play important roles in Swedish science. In 1982, his remarkable skills were recognized on the European level by his appointment as the second Director General of the European Molecular Biology Laboratory (EMBL), which he ran until 1993. There he established a balance between work on instrumentation and on scientific objectives, insisting that most group leaders stay for only a fixed time so as to generate a constant renewal of the staff. It was an inspired policy: many of the leading scientists throughout Europe got their start at EMBL. He also redirected the scientific focus toward cellular and developmental biology. Among his many innovations at EMBL, Lennart realized that computation was becoming an important part of biomedical research and opened an outstation for biocomputation that has been a major European window on the growing world of genomic research.

In 1993 Lennart returned to the US to be the Founding Director of the Skirball Institute of Biomolecular Medicine at the New York University School of Medicine. He set high standards for recruitment and brought a very strong group to Skirball. He spent five years in New York, setting up Skirball's structure and establishing it as an important unit within NYU. Many of the people he recruited are still at Skirball; he set them on a trajectory of accomplishment and leadership. Today, in fact, one of his recruits, Ruth Lehmann, is its director. This legacy is a notable example of what can be accomplished in a semi-independent institute within a larger academic structure.

After leaving Skirball, Lennart returned to Stockholm, Sweden, where he joined the Karolinska Institute and was Emeritus Professor of Microbiology there when he passed away. He stayed active in science and was an author on a publication in 2011 describing the consequences of a knockout of a receptor shared by coxsackieviruses and adenoviruses. It was a protein he had discovered almost 15 years earlier.

He had been a foreign member of the US National Academy of Sciences since 1992 and, of course, was a member of the Royal Swedish Academy of Sciences. He consulted with many companies, notably Astra in Sweden. He had many honorary degrees.

Yet, science was not Lennart's only passion. He was devoted to his family. His wife, Malin, was a partner in all the challenges Lennart took on and remains a gracious friend to all of Lennart's associates. His boys were the apple of his eye and their successes in life were a source of great pride. Swedes know how to do summer, and for Lennart and Malin, their summer home was a key release from the pressures of busy lives. Lennart was an excellent sailor; he even spent two years at sea when he was young. He and Malin, along with my wife and I and many other friends, rented sailing boats around the world. Our most remarkable time was sailing in the northern islands of the Kingdom of Tonga, in the South Pacific. Lennart was a remarkable captain who let others run the ship until that moment when leadership and experience were needed—then he took over and got the ship through the tough patch.

All who knew Lennart will remember him with a pipe in his mouth, furiously smoking the Prince Albert tobacco he imported from the US. He would leave residues of his tobacco on the sides of the boats we sailed because he tamped out the ashes of used tobacco on the boat's gunwale.

Lennart was a rare and irreplaceable person, a true individual. His science was path-finding, his leadership strong and imaginative, his friendship deep. He will be missed by all who knew him.

***Editor's note*** **:** The editors at *PLoS Biology* recognize the life and work of Lennart Philipson and are grateful for his years of service on the journal's editorial board.

